# Conservation Evolutionary Biology: A Unified Framework Connecting the Past, Present, and Future of Biodiversity Conservation

**DOI:** 10.1093/molbev/msaf122

**Published:** 2025-05-30

**Authors:** Yisi Hu, Wenliang Zhou, Yibo Hu, Fuwen Wei

**Affiliations:** Center for Evolution and Conservation Biology, Southern Marine Science and Engineering Guangdong Laboratory (Guangzhou), Guangzhou, China; Center for Evolution and Conservation Biology, Southern Marine Science and Engineering Guangdong Laboratory (Guangzhou), Guangzhou, China; CAS Key Laboratory of Animal Ecology and Conservation Biology, Institute of Zoology, Chinese Academy Sciences, Beijing, China; Center for Evolution and Conservation Biology, Southern Marine Science and Engineering Guangdong Laboratory (Guangzhou), Guangzhou, China; CAS Key Laboratory of Animal Ecology and Conservation Biology, Institute of Zoology, Chinese Academy Sciences, Beijing, China; Jiangxi Provincial Key Laboratory of Conservation Biology, College of Forestry, Jiangxi Agricultural University, Nanchang, China

**Keywords:** conservation evolutionary biology, endangered species, adaptive evolution, population genomics, conservation practice

## Abstract

Evolutionary insights are fundamental to biodiversity conservation, as they reveal the ultimate causes that drive adaptation and persistence of populations, species, and ecosystems over time. In recent decades, the field of conservation biology has expanded to incorporate species' evolutionary histories, adaptive mechanisms, rapid evolution, and interactions with ecosystems to better understand the complexities of species' persistence in the long run. To address this growing recognition that conservation is inherently an evolutionary question, we propose “conservation evolutionary biology” as a new subdiscipline of conservation biology. It aims to explore the past, present, and future of biodiversity from an evolutionary viewpoint, revealing how species and ecosystems respond and adapt to changing environments, thereby to inform science-based, future-oriented conservation strategies to sustain biodiversity in a rapidly changing world. This article highlights the principles, research focus, methodologies, and practices of this emerging field, encouraging further exploration to address the conservation challenges of our time within a unified framework.

## Introduction: Considering Conservation Biology in the Lirtight of Evolution

Biodiversity, a cornerstone of our planet's health, is at risk (Pimm et al. 2014). In response to this global challenge, conservation biology emerged in the late 1970s with a mission to safeguard endangered species and comrbat the alarming loss of biodiversity. Traditionally rooted in ecology, population demography, and animal behavior and physiology, this young discipline initially focused on addressing the “how” questions surrounding the mechanisms of biodiversity loss, species extinction, and assisted recovery, such as identifying the intermediate drivers behind population declines, designing reintroduction projects for population recovery, and constructing protected areas to cover habitats vital for population persistence (Soulé 1986).

Yet, these conservation efforts have yielded both exciting successes and disheartening failures, mirroring the historical rise and fall of countless species in the natural world. This keeps reminding us that understanding the “why” questions, which unveil the ultimate causes behind observed phenomena of species adaptation and interaction with environment, is crucial. Indeed, the multidimensional diversity observed in ecosystems are products of complex evolutionary dynamics over deep timescales. We cannot describe and understand the pattern of biodiversity without knowing the evolutionary process of its formation. Moreover, the very purpose of conservation is to combat species extinction and ensure the long-term persistence of biodiversity, which inherently involves understanding the evolutionary trajectories of species, how species maintain their evolutionary potential over time and continue to survive in a changing environment. Neglecting these evolutionary aspects in conservation practices can lead to serious problems, such as evolutionary mismatch, loss of genetic diversity and local adaptation, and reduced resilience to fast-changing environments, ultimately compromising species' potential survival in the future.

**Fig. 1.**
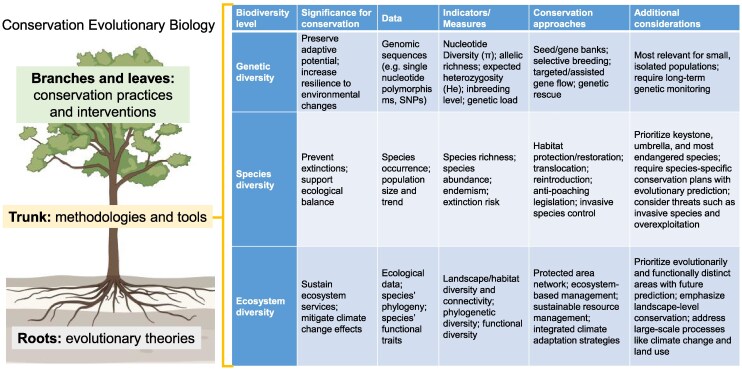
A conceptual framework of conservation evolutionary biology and its application to biodiversity conservation. This figure illustrates how conservation evolutionary biology is rooted in evolutionary theories, supported by specialized methodologies and tools, and translated into practical conservation strategies across genetic, species, and ecosystem levels.

Thus, recognizing the evolutionary nature of conservation practices is not just a theoretical consideration, but a practical necessity for ensuring the effectiveness and long-term sustainability of conservation efforts. Studies are now addressing how biodiversity responds to evolving threats whose impacts span years to decades and centuries. They go beyond the traditional fields of conservation biology, looking into the mechanisms that shape genetic diversity, drive speciation, and confer resilience in the face of environmental disturbances. Nonetheless, evolutionary studies in conservation biology have been scattered for a long time, and few scholars have systematically studied it as a branch of conservation biology (Ferrière et al. 2004). Building on our earlier proposal to establish a new subdiscipline of “conservation evolutionary biology” that integrates evolutionary principles and methodologies into addressing conservation challenges (Wei 2018; Wei et al. 2019), we further elaborate and expand this framework to unify research on the past, present and future of biodiversity conservation, in order to provide a scientific basis for designing forward-looking conservation strategies. Here, we introduce and discuss the key research themes, methodologies and practical applications of conservation evolutionary biology based on the latest scientific advances, and advocate for continued development of the field to address modern conservation challenges (Fig. 1).

## Understanding the Past: How the Past Evolutionary Forces Shape the Current Pattern of Biodiversity?

The current pattern of biodiversity is deeply influenced by past evolutionary forces that have acted on genetic materials, species, and ecosystems over time. Investigating these forces provides essential insights into the present distribution, traits, and ecological relationships of living organisms and their conservation needs.

### Evolutionary History and Endangerment Process

Evolutionary forces impact species through population divergence, speciation, extinction, hybridization, etc., shaping species' population structure and distribution range we observe today. Understanding these evolutionary histories is essential for delineating population, subspecies, species, and even hybrid to define the entity of a conservation unit. This is a fundamental step for biodiversity assessment and legislation, as it determines how resources are allocated and directly influences the effectiveness of conservation efforts (see Box 1). In the critically endangered Chinese giant salamanders, misguided *ex situ* propagation programs used to release nonlocal and even artificial interspecific hybrid individuals into the wild, before the identification of several cryptic species in this species complex based on their evolutionary relationship (Yan et al. 2018). So far, only 1 genetically pure and self-sustaining population of Chinese giant salamanders is found in the wild, representing a precious opportunity to rejuvenate this newly identified species (Chai et al. 2022). With whole-genome data and the constantly updating genomics tools, complex evolutionary history could be reconstructed to infer fluctuations in effective population size, population divergence, and gene flow over time (Bourgeois and Warren 2021). Research on the endangered red pandas shows that the population from middle Himalaya has experienced 3 bottlenecks, resulting in low genetic diversity and high genetic load, while 3 populations in the east of the Yalu Zangbu River in China and Myanmar have experienced 2 bottlenecks but 1 large expansion with higher genetic diversity within populations. These results, together with other genomic and morphological evidence, indicate the existence of 2 phylogenetic species of red pandas (*Ailurus fulgens* and *A. styani*) with distinct histories, distributions, and current statuses (Hu et al. 2020). These new findings underscore the urgent need to reassess conservation management of red pandas, both in the wild and in captivity.

Box 1 **Species, hybridization, and Evolutionary Significant Units for conservation**

In most conservation frameworks, such as the IUCN Red List, the conservation unit under consideration is typically species. However, species delimitation has long been complicated by varying definitions, resulting in both underestimation of species diversity and also the issue of “taxonomic inflation.” Taxonomic inflation refers to an excessive proliferation of species delimitations without robust genetic, ecological, or reproductive evidence, which can distort conservation assessment in both species count and endemicity representation (Isaac et al. 2004). In some cases, hybridization between species or populations further complicates conservation decisions regarding whether hybrids and the hybridization process should be preserved. Hybridization can have contrasting effects: while it may threaten the genetic integrity and distinctiveness of rare species through genetic swamping, it can also introduce beneficial genetic variation and revive declining populations. Such effects have been observed in adaptive radiations, where hybridization between species contributes to increased genetic diversity and facilitates rapid radiations (Grant and Grant 2019). Thus, these conservation challenges are intrinsically evolutionary questions and require an evolutionary perspective to address effectively.

From an evolutionary standpoint, managing species delimitation and hybridization involves prioritizing lineages that represent distinct evolutionary trajectories and possess genetic attributes critical for long-term survival. The evolutionary significant unit (ESU) framework provides an evidence-based and applicable approach to defining conservation units, emphasizing genetic and ecological differentiation rather than taxonomic ranks (Hoelzel 2023). Genomic data (e.g. neutral nuclear markers) can help identify distinct evolutionary lineages by revealing independent demographic trajectories, significant genetic divergence, and restricted gene flow (see main text). Ecological evidence on distribution ranges, habitat preferences, feeding, and reproductive behaviors further distinguishes genuinely isolated evolutionary lineages with distinct traits and conservation needs from taxonomically split taxa lacking divergence.

For instance, in staghorn corals (*Acropora*), one of the most dominant reef-building coral genera in the world, over 400 species have been described, mostly based on morphology, but only 120 to 160 are considered valid (Veron et al. 2025). The IUCN Red List currently recognizes 150 *Acropora* species, with 110 classified as threatened and another 32 as data deficient (IUCN 2025). Given the wide distribution and highly variable phenotypic traits of *Acropora* species, large-scale genomic, ecological, and reproductive studies applying the ESU principles are essential to both refining taxonomy and species boundaries and distinguishing isolated local populations under high risk of silent extinctions (Richards et al. 2016). This can lead to the identification of robust ESUs below the species level with reliable differences in geographic distribution, ecological traits (e.g. depth), and genetic distinctiveness. Shifting to such an ESU-based framework helps appropriately describe and adequately conserve the diversity of species without over-splitting taxa based on subtle differences in morphology, thus ensuring more accurate conservation assessment and management for these ecologically important but vulnerable species.

Conversely, hybridization reflects the evolutionary continuum of species and populations, sometimes leading to lineage merging (i.e. species or population collapse) rather than splitting. As a natural process, hybridization is not inherently detrimental, and its conservation implications depend on the evolutionary and ecological consequences. A recent study reports that a threatened species, the Peruvian fur seal, has originated from hybridization of the South American fur seal (*Arctocephalus australis*) and the Galapagos fur seal (*A. galapagoensis*) and suggests considering it as a full species for independent conservation (Lopes et al. 2023). Among the Caribbean *Acropora* corals, *A. palmata* and *A. cervicornis* naturally interbreed to produce the hybrid *A. prolifera*. Research suggests that *A. prolifera* has persisted in the wild with high abundance comparable to the parental species in some reefs, exhibiting better survival, faster growth, and even enhanced adaptation to extreme habitats (VanWynen et al. 2021). Despite additional evidences suggesting that *A. prolifera* has existed in the late Pleistocene and that individuals can live for thousand years through asexually reproduction, its protection is still under debate (Precht et al. 2019). To date, unlike the 2 parental species that are both classified as “Critically Endangered” on the IUCN Red List and listed as “threatened” under the US Endangered Species Act, *A. prolifera* is not included due to its hybrid status. In an evolutionary perspective, the coexistence of *A. palmata*, *A. cervicornis*, and the hybrid *A. prolifera* could be a snapshot of the ongoing speciation of 3 different species before they develop more rigorous reproductive isolation, or the upcoming collapse of the 2 parental species due to continuous hybridization and backcross. These evolutionary processes will continue to take place in the wild, and may accelerate and intensify due to species range shifts in changing environments. While practical issues remain to include hybrids in legislation, scientists are actively advocating for its independent conservation and potential use in reef restoration, given its role in evolutionary processes and its ability to provide valuable habitat in declining coral reef ecosystems (Precht et al. 2019; VanWynen et al. 2021).

In some cases, controlled hybridization can serve as a tool for genetic rescue, introducing adaptive genetic variation in small populations facing extinction. Some conservation biologists thus encourage a shift toward a web-of-life framework, which embraces species with admixed ancestry or complex evolutionary origins in conservation efforts to foster long-term adaptation (vonHoldt et al. 2018). However, when hybrids typically display phenotype-environment mismatch or when hybridization leads to reduced population growth and loss of adaptive genetic variations of parental species—such as an endangered native species hybridizing with an expanding invasive species introduced by human activities—management interventions are necessary to prevent the loss of unique genetic and phenotypic adaptations in native species.

Integrating evolutionary principles into species definitions allows for reasonable delineation of conservation units (i.e. ESUs) and ensures that conservation managements such as captive breeding and habitat corridor construction are not misguided, and that conservation efforts are directed toward maintaining the evolutionary processes sustaining ecological functions and biodiversity at both intraspecific and interspecific levels, rather than being constrained by taxonomic debates.

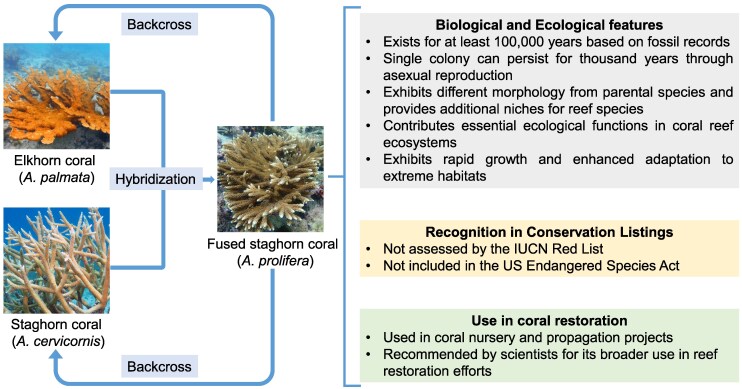

**Inserted figure in Box 1. Hybridization and conservation of *Acropora* corals in the Caribbean.** This figure illustrates the natural hybridization between *A. palmata* and *A. cervicornis*, resulting in the formation of *A. prolifera*, which can also backcross with its parental species. While both parental species are considered endangered and receive conservation attention, *A. prolifera* is not currently protected despite its distinct traits and promising potential for use in reef restoration. Photo credit: Pauline Walsh Jacobson, CC BY 4.0 (elkhorn coral and staghorn coral); Donají Graham, CC BY-NC 4.0 (fused staghorn coral).

Evolutionary events also intertwine with geographical change, climate shifts, and human activities to influence species' movement across landscapes and their persistence in the environments. For instance, sea level rise and volcanic activity over the past 10 million years may have contributed to habitat loss and fragmentation for the endangered Malagasy baobabs, leaving lasting impacts on their restricted present-day distributions along western and northern Madagascar (Wan et al. 2024). Similarly, genomic analyses suggest that the endangered mangrove species *Pellacalyx yunnanensis* became stranded in unfavorable habitats during dramatic climate shifts and faced reproductive challenges due to the loss of seed dormancy traits. These factors together have led to its historically small population and narrow contemporary distribution (Shao et al. 2025). A study on 263 bird species also suggests that morphological and life history traits may have influenced the response to climate warming and cooling over the past million years, which helps identify species more sensitive to environmental changes and guide their conservation practices (Germain et al. 2023). Interestingly, over the past tens of thousands of years, ubiquitous declines are found in world's megafauna across landmasses, which are believed to be driven by worldwide expansion of *Homo sapiens* instead of changes in climate (Bergman et al. 2023). Recognizing how different past events impact species is thus essential for understanding species' endangerment process and prioritizing conservation actions, especially for species that have shown sensitivity to past environmental changes and anthropogenic influence.

### Adaptation and Coevolution in Specific Environments

In contrast to species that fail to adapt to environmental changes and face extinction, some species gradually evolve by adjusting their physiological, behavioral, and morphological traits, enabling them to thrive in shifting environments and occupy specific ecological niches (Hu et al. 2023a). Understanding these adaptations is crucial for designing conservation strategies that meet a species' unique ecological requirements, and therefore a main component of conservation evolutionary biology research. The morphological, ecological, physiological and genomic evidence of diet adaptation of the giant panda serves as an excellent example to show that species have striking ability to respond and adapt to environmental changes coordinately in different aspects, securing their own persistence and flourishment (Nie et al. 2015; Wei et al. 2015; Rudolf et al. 2022). The critically endangered orangutans from North Sumatra (*Pongo abelii*) and Borneo (*P. pygmaeus*) display different adaptive responses to their own islands during the past million years. The Sumatran orangutans have evolved in cognition and behavior associated with higher degrees of sociality, while the Bornean orangutans evolved in physiological adaptations in response to fluctuations of food supplies (Mattle-Greminger et al. 2018). These results could serve as the foundation for targeted conservation practices for these closely related but divergently evolved species. With the arrival of the omics era, genome-based science is increasingly providing valuable insights into the adaptive mechanisms of endangered species. A series of studies report that species living in extreme environments, such as plants in high-altitude habitats (Zhang et al. 2023), mangroves in land–sea interface (He et al. 2020), and wild fish in polluted water (Reid et al. 2016), often exhibit convergent adaptation by altering similar genetic pathways, which reveals key genomic mechanisms of adaptation and offers critical support for species conservation. The development of Telomere-to-Telomere (T2T) genomes, along with initiatives like the digital Noah's Ark (a repository of T2T genomes for endangered species) thus holds great promise for preserving and potentially restoring the biodiversity of endangered species (Wei et al. 2022).

In addition, species often interact and coevolve with one another, leading to reciprocal adaptations. These coevolutionary relationships, such as those between mutualistic symbiotic partners, competitors, hosts and parasites, as well as predator and prey, also shape species traits and ecological roles. Changes in one species can have cascading effects on others, influencing community structure and biodiversity patterns (Dehling et al. 2022). The reef-building corals in shallow waters host a group of algae (Symbiodiniaceae) within their cells to provide themselves with photosynthetically fixed carbon as nutrients, which is fundamental to the development of coral reef ecosystem. Corals and algae have evolved interdependent metabolic mechanisms through gene loss and horizontal gene transfer (Shinzato et al. 2011, 2021; Shoguchi et al. 2018), making them closely interconnected in the face of environmental threats. Additionally, corals can select and even foster heat-tolerant algae strains best suited for prevailing environmental conditions to increase their own bleaching tolerance (D’Angelo et al. 2015; Buerger et al. 2020). These findings provide valuable knowledge about species vulnerability and response to environmental stress and call on systematic conservation of “coral holobionts” instead of the coral animals alone (Hu et al. 2021a). In wild mammals, emerging studies are now applying metagenomics to study coevolution between the host and microbiota. For example, giant pandas host specific bacteria in their gut to degrade cellulose from their bamboo diets and adapt to changing nutrient supplies in different seasons (Zhu et al. 2011; Wu et al. 2017; Huang et al. 2022; Hu et al. 2024). The functional differences in microbial compositions reflect the host's physiological status and can serve as indicators of the reintroduction process and the success of releasing captive giant pandas into the wild (Huang et al. 2023). The relationships between plants and pollinator (e.g. figs and fig wasps; orchids and moths) or seed dispersers are also products of coevolution, reflecting a dynamic evolutionary equilibrium. When these specialized interactions break down, they are not easily compensated, threatening the persistence of many endangered species and triggering cascading ecological and economic consequences (Wei et al. 2021; Mendes et al. 2024). In general, these coevolutionary relationships not only pose challenges to one-sided conservation for target species, requesting more orchestrated conservation planning, but also provide valuable opportunities to improve the current conservation practices via systematic management of the interacting organisms.

### Phylogenetic Diversity Conservation of Community

Biodiversity patterns at the community or ecosystem level reflect the evolutionary history of the region shaped by species interactions and dynamics over time. Measurement of phylogenetic diversity based on the evolutionary distances of sympatric species (Faith 1992; Clarke and Warwick 1998) could expand the focus from the evolutionary distinctiveness of individual species to a community-wide perspective. Different phylogenetic diversity metrics characterize the richness, divergence, and regularity of phylogenetic relationships in a given community, which helps understand fundamental questions about community assembly, speciation, and endemicity, niche space occupancy and community stability, ecosystem resilience and functionality (Tucker et al. 2017). In practice, phylogenetic diversity metrics help identify the “evolutionary cradle” (generating more young species) and “evolutionary museum” (preserving more old lineages) that represent different processes of species evolution (Stebbins 1974). Typically, communities composed of more distantly related species (higher phylogenetic divergence) possess greater evolutionary potential and resilience to environmental changes. Phylogenetically distant species are also more likely to display higher trait diversity and functional differentiation, contributing to a broader range of ecosystem functions and services. Consequently, phylogenetic diversity represents a unique perspective for identifying conservation priorities by explicitly linking biodiversity to ecosystem function and stability.

Emerging studies now integrate genetic diversity, species diversity, and phylogenetic diversity to identify biodiversity hotspots for global conservation planning. In tropical Africa, researchers found a strong correlation between plant genus richness and phylogenetic diversity, with high degree of overlap between centers of phylogenetic endemism and existing protected areas, suggesting that current conservation efforts effectively cover plant biodiversity (Dagallier et al. 2020). However, a global study reports incongruence among phylogenetic diversity hotspots and species diversity hotspots of terrestrial vertebrates and angiosperms, as well as insufficient coverage by the existing protected areas network (Daru et al. 2019). A study on marine animals suggests that safeguarding 22% of the ocean based on the combination of species, genetic, phylogenetic diversity metrics could effectively conserve 95% of currently known marine biodiversity (Fan et al. 2023). Emerging regional, continental, and global studies that integrate multiple dimensions of biodiversity are accumulating knowledge to identify evolutionary hotspots and refuges across diverse taxa, providing additional insights into the irreplaceability of communities and conservation priority by considering the underlying ecological and evolutionary processes (Lu et al. 2018; Hu et al. 2021b; Luo et al. 2024; Omollo et al. 2024). Such holistic approach could enhance spatial conservation planning and support progress toward the “30×30” conservation target (CBD 2022).

Overall, by unraveling the outcomes of evolutionary forces through up-to-date techniques, we have gained valuable insights into the evolutionary history, adaptive strategies, ecological interactions, vulnerabilities, and uniqueness of species. This understanding forms the foundation for conservation efforts that aim to preserve and sustain the biodiversity shaped by the dynamic interplay of evolutionary processes over time.

## Monitoring the Present: Unveiling Species Adaptive Responses to Environmental Changes

**Fig. 2.**
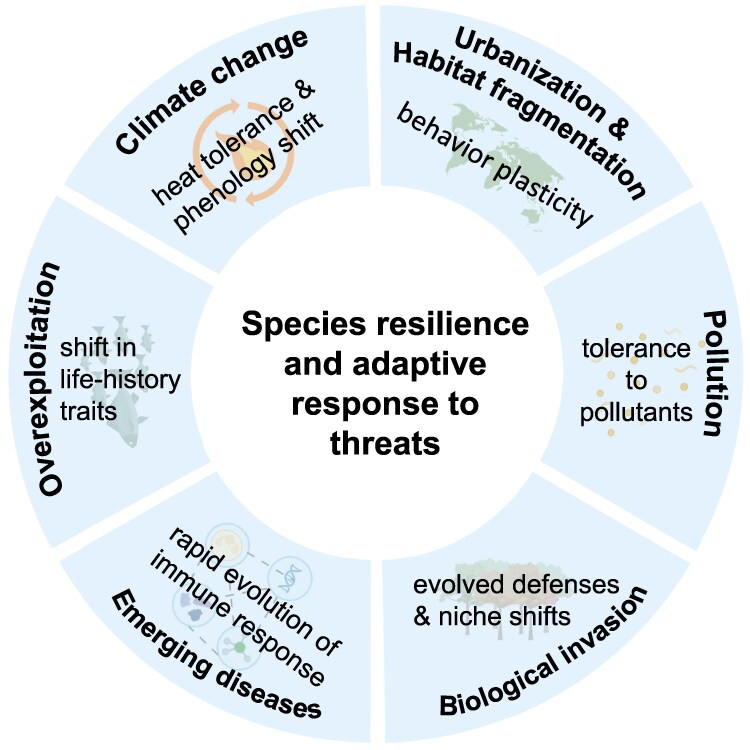
The mechanisms of species' resilience and rapid adaptation in mitigating environmental threats.

In the era marked by rapid environmental changes and extensive human disturbance, a vital focus of conservation biology lies in understanding the mechanisms that underscore species resilience and their adaptive responses to presently evolving environmental threats. This boosts the exploration of phenotypic flexibility, rapid adaptation, and the intricate interplay between species and their environment. This accumulating knowledge can be applied in conservation management of biological invasions, disease outbreaks, human overexploitation, etc., and may have a chance to transform our current practice (Fig. 2).

### Molecular Mechanisms of Adaptive Responses

A wealth of evidence, derived from studies on both laboratory organisms and wild populations, has reported species' remarkable ability of rapid adaptive responses of morphological and physiological traits to environmental changes (Duncan et al. 2020; Mallard et al. 2020). During recent adaptation to new habitats, an amphibian species is shown to diversify its dispersal and movement behaviors to promote adaptive divergence of the population into habitat-dependent, genetically differentiated subpopulations (Hendrix et al. 2017). Accumulating evidence proves that diversification of plastic traits can serve as a stepping stone toward adaptation to new and changing environmental conditions in many natural populations (Snell-Rood et al. 2018), linking phenotypic flexibility and rapid evolution from the timescale of individual's lifespan to multiple generations and from the scope of single individual to the entire population. In the past 2 decades, advanced omics techniques have aided in capturing the underlying mechanisms of organisms' phenotypic flexibility and rapid evolution in response to various threats in diverse taxa, including gene expressional changes, genetic variability, and epigenomic modifications (Leung et al. 2022; Bonnet et al. 2022; Hackerott et al. 2023; Walter et al. 2023). Meanwhile, environmental changes can induce genetic variability (such as through the activation of transposable elements) and trigger rapid evolutionary processes by selecting the most advantageous variations for survival (Pimpinelli and Piacentini 2020). It is also important to distinguish phenotypic plasticity and rapid adaptation, as it may sometimes hinder genetic adaptation if plastic responses reduce the strength of selection pressure that drives genetic change. Conservation efforts thus need to carefully consider the balance between leveraging phenotypic plasticity for immediate benefits and promoting conditions that allow for adaptive genetic evolution in the long run.

### Understanding Species' Responses to Changing Environments

Climate-related local extinctions have already been documented in hundreds of species, with higher frequencies observed in tropical species, animals, and freshwater habitats (Wiens 2016). Understanding species' responses to environmental changes is particularly critical in the context of global climate change, as it sheds light on how adaptive evolution may mitigate the negative impacts of changing environments. Thermal acclimatization experiments have been widely used to study species' heat tolerance and rapid adaptive responses at genetic, epigenetic, and other levels (Schaum et al. 2018; Perez and Aron 2020; Stager et al. 2021), exploring the extent to which species can survive and adapt the thermal conditions, with potentials to inform habitat management strategies and assisted evolution programs for vulnerable wild species. Global climate change is also influencing life in the water, with ocean acidification posing severe challenges for marine organisms that depend on calcium carbonate for their skeletons and shells. Corals, for instance, are coping with acidification by fine-tuning gene expression through DNA methylation changes and forming more porous skeletons with reduced calcification (Liew et al. 2018). These epigenetic modifications not only provide short-term survival benefits but may also influence long-term evolutionary outcomes if they persist across generations and interact with genomic inheritance (Haghani et al. 2023; Hu et al. 2023b). Supporting this, experiments on wild sticklebacks demonstrates that inducible epigenetic marks facilitate transgenerational plasticity in response to salinity changes, allowing populations to adapt to new environments along a salinity gradient (Heckwolf et al. 2020). In addition, species subjected to other threats, such as overexploitation, can exhibit rapid evolutionary responses including faster growth, earlier maturation, or increased reproductive output, aiding recovery under intensive harvesting (Heino et al. 2015). Taken together, these findings suggest that phenotypic flexibility and rapid evolution in wildlife in response to changing environments may be more widespread and faster than previously anticipated. Yet, without proactive conservation strategies, these adaptive responses might not be sufficient to guarantee species' persistence, as they may track a shifting optimum while consistently lagging behind (Radchuk et al. 2019).

On the other hand, cities, as the carrier of human settlements, are one of the concentrated areas of interactions and conflicts between man and wild species. Urbanization has resulted in dramatic environmental changes, including increased temperatures, more impervious surface cover, altered hydrology, and elevated pollution. As a result, city-dwelling species may shift their daily activity patterns (Gaynor et al. 2018) and behavior modes (Atwell et al. 2012), and increase tolerance to urban heat islands (Campbell-Staton et al. 2021) and pollutions (Reid et al. 2016) through contemporary adaptive evolution. Nonetheless, cities usually elevate genetic drift and restrict gene flow, resulting in genetic differentiation between populations and even extinction in various taxa (Johnson and Munshi-South 2017; Roux et al. 2019). Therefore, it is crucial to understand the adaptive and nonadaptive responses and evolution associated with urbanization, and how these processes affect organismal survival and coexist with human in the urban areas. For instance, urban lizards, compared to forest lizards, can reduce maladaptive heat-induced plasticity and increase adaptive plasticity, resulting in greater heat tolerance to live in the city (Campbell-Staton et al. 2021). Such research provides valuable opportunity for the conservation of wild species adapted to urbanization, and gives us a chance to manage the wild populations to facilitate the establishment of sustainable urban ecosystems.

### Conservation Applications Related to Biological Invasion and Disease Outbreaks

The knowledge of adaptive response is also valuable for conservation management of biological invasions and could be actively integrated into landscape management and multispecies management. The purple loosestrife (*Lythrum salicaria*), an invasive plant species in North America, has rapidly evolved to adapt local environments along a 1000-km climatic gradient within 100 year, with the northern population evolved earlier flowering at a smaller size and the southern population evolved later flowering at a larger size (Colautti and Barrett 2013). Adaptive rapid evolution of flowering time in another invasive plant is found to be driven by recent transposable element insertions at *FLOWERING LOCUS C* (*FLC*) that affect mRNA stability and gene expression levels (Niu et al. 2019). Similarly, a decade of research on the invasive cane toads (*Rhinella marina*) in Australia has identified their changes in critical thermal maxima in high cold montane regions through fast-acting phenotypic plasticity as well as inheritable shifts in gene expression related to immune responses of long-distance dispersal (Rollins et al. 2015). Reduced levels of DNA methylation and increased levels of gene expression variability also play a role in invasive invertebrate populations to promote phenotypic plasticity and thus facilitate expansion (Ardura et al. 2017; Gatzmann et al. 2018). These findings emphasize the importance of explicitly considering that invasive species are not static entities, but can present phenotypic variation through epigenetic changes and gene expression re-programming, or even rapidly evolve novelty to occupy a broad range of habitats, with important implications for controlling their future spread. Native species, in turn, must also adapt to this dynamic threat by evolving comparable rapid responses or exhibiting phenotypic plasticity to withstand competition and environmental shifts brought about by invasions.

Epidemic disease is another threat faced by global biodiversity and its transmission can act as selective pressure to drive rapid changes in population dynamics and genetic structure of wild populations. Rapid and parallel evolution of disease resistance has been observed in multiple species exposed to lethal pathogens. Rabbit populations in Australia, France, and the UK all developed increased resistance to the pandemic myxoma virus over 60 year, partly through nonsynonymous mutations in several immunogens (Alves et al. 2019). Similarly, natural populations of amphibians exposed to the *Batrachochytrium dendrobatidis* (Bd) pathogen causing chytridiomycosis (Voyles et al. 2018) and bats facing white-nose syndrome caused by *Pseudogymnoascus destructans* (Langwig et al. 2017) have shown signs of ongoing evolution in disease resistance. Some wildlife species adopt another strategy that does not lower the infection intensity but enhances the disease tolerance to the emerging pathogens. Such cases include the evolution of increased infection tolerance to parasites via extensive fibrosis after marine sticklebacks colonizing freshwater lakes (Weber et al. 2022) and the rapid evolution of pathogen tolerance facilitated by targeted immune response in house finches within 25 year (Henschen et al. 2023). The rapid evolution of disease resistance and tolerance in natural populations offers the hope that species will persist even in the face of deadly pathogens. Meanwhile, the pathogen itself can quickly evolve in pathogenicity, infectivity, and host specificity. Understanding the mechanisms underlying the rapid evolution and coevolution of hosts and pathogens thus promises to inform targeted and systematic management strategies in species of conservation concern as well as safeguard the ecosystems and public health (Xu et al. 2025).

Integrating evolutionary biology with molecular genetics, wildlife physiology, and behavior ecology is needed to theoretically and practically guide conservation planning and foster dynamic, multispecies, and ecosystem-based management tailored to evolving conditions. These collaborative efforts across disciplines are essential to support real-world practices and optimize conservation strategies, promoting more proactive interventions in the face of environmental challenges.

## Safeguarding the Future: Designing Future-oriented Conservation Practices With Evolutionary Insights

The growing understanding of the molecular mechanisms underlying species' evolutionary dynamics suggest that species also have potential to respond to future environmental change by expanding their current niche and adapting to new conditions. These evolutionary processes can be predicted with greater accuracy coupled with the accumulations of evolutionary data and advances of genomic tools. Integrating such evolutionary predictions into conservation strategies holds the promise to enhance conservation outcome for the long-term persistence of target species and ecosystem under changing environment.

### Improving Species' Range Shift Predictions by Incorporating Evolutionary Genomics

Species distribution model (SDM) that relates species' occurrences to environmental variables is an essential conservation tool for predicting potential range shifts under climate change scenarios. Some improved models have considered population abundance (Ehrlén and Morris 2015), species tolerance or physiological limits (Martínez et al. 2015) and biotic interactions (Poggiato et al. 2021). However, these models basically assume species to be an unchanging entity and fail to account for species' evolutionary processes and adaptive potential, thus leading to overly pessimistic predictions. Novel approaches that incorporate genetic variation and population dynamics can account for species' adaptive potential by simulating evolutionary processes and optimums of key biological and physiological traits. These hybrid SDMs or eco-evolutionary models predict evolution in the future based on ancestral state (with space-for-time or time-for-time approaches) and evolved state with genomic data (Waldvogel et al. 2020). These studies usually report less projected range losses or population declines when taking into account of evolution (Bush et al. 2016; Benito Garzón et al. 2019; Razgour et al. 2019). For instance, species in high latitude are predicted to evolve thermal tolerance, but only under mild warming scenarios and unable to persist under more severe climate change scenarios (Bay et al. 2017). Observed limited genomic variations associated with adaptation also predict population declines due to failure to keep pace with future changes, such as for the “genetically vulnerable” yellow warbler populations (Bay et al. 2018). Exposito-Alonso et al. combined extensive genome information and field experiments under different climate conditions of over 500 natural *Arabidopsis* lines and modeled climate-driven selection based on genomic signatures of local adaptation. They predicted an increase in directional natural selection under future climate change, which may put many native populations at evolutionary risk (Exposito-Alonso et al. 2019). These cases together show that evolution-informed models could deliver more realistic prediction of species distribution and extinction risk, which may alter our understanding of species vulnerability to changing environment.

Efforts to integrate evolutionary dynamics under future conditions and predict species' fate are still expanding, with more evolutionary factors such as drift, gene flow, population dispersal, and genetic load being considered in an integrated framework to capture the complexities of evolution (Aguirre-Liguori et al. 2021; Yamamichi et al. 2023). However, several challenges remain for the emerging research. A key difficulty lies in gathering empirical data, as obtaining high-quality genetic and ecological data from natural populations across landscape or timescale is labor-intensive and costly. This could be mitigated in controlled environments like common garden experiments to parameterize the models; however, such experiments are often unfeasible for rare and endangered species, where data for robust analyses is usually insufficient. In addition, when interpreting genomic and ecological data, it is sometimes argued that not all genetic and phenotypic variations that arise in response to changing environments are beneficial (Chapuis et al. 2021; Mundinger et al. 2023), adding even more uncertainties to establish the link between genetic changes and long-term adaptation. Systematic long-term research projects, though still relatively rare, are filling this gap to capture evolutionary dynamics that are otherwise difficult or impossible to detect through short-term observation (Stroud and Ratcliff 2025). Overall, the push to incorporate evolutionary insights into conservation modeling is gaining more attention, but it requires overcoming significant theoretical and technical challenges to ensure that predictive models are both accurate and applicable across diverse species and ecosystems.

### Forecasting the Outcomes of Conservation Interventions by Incorporating Evolutionary Principles

In more severe conditions when small population could no longer persist in the long-term, conservation interventions such as genetic rescue and translocation are needed to save its fate from going to extinction. Ensuring the long-term persistence of this population requires understanding of the match or mismatch between phenotypes (morphological, physiological, and behavioral) and the underlying genotypes and epigenotypes in relation to the environment. This involves evaluating genetic load (the burden of deleterious alleles), genomic offset (the risks of maladaptation under changing conditions), and other potential evolutionary responses that could either support or hinder conservation goals. Iterative forecasting models applying evolutionary genomics to translocation projects are developed to guide conservationists from preparation stage to post-translocation monitoring and forecasting (Seaborn et al. 2021). Evolutionary forecasting can also predict the potential benefits and risks of introducing new genetic material into inbred small populations. In an endangered bird species *Zosterops silvanus*, hybridization with its congener may enable the greatest long-term population growth and evolutionary rescue, as the targeted gene flow rapidly provides genetic variability to the small populations and guarantees more adaptive evolution in the future (Vedder et al. 2022). Emerging research and tools based on whole-genome data have made it possible to identify and choose advantageous admixture for conservation, and to conserve taxa that have experienced or are experiencing gene flow and introgression (Taylor and Larson 2019).

**Fig. 3.**
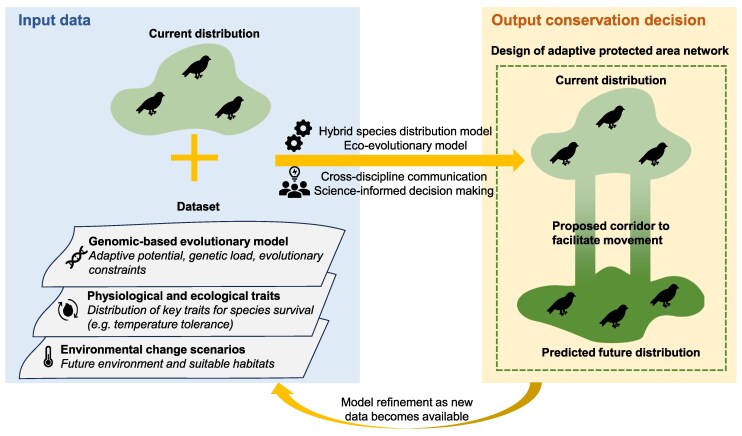
Evolution-informed models for predicting species distribution and prioritizing conservation efforts. By combining genomic-based evolutionary models, key physiological and ecological trait data, and environmental change projections, we can predict shifts in trait distribution and suitable habitats. Overlaying these predictions with species' current distributions enables the development of hybrid SDMs or eco-evolutionary models. These models can inform the design of adaptive protected area networks by identifying future habitats and ecological corridors, thus supporting species movement in the landscape and enhancing long-term conservation under changing environmental conditions.

Apart from conservation interventions directly acting on living organisms, other strategies such as protected area construction and habitat restoration can also benefit from evolutionary insights. Modeling the adaptive potential of species and identifying evolving environmental thresholds where they might struggle to persist, has the potential to guide future conservation (Fig. 3). This includes delineating conservation units based on geo-genomic simulations, prioritizing protected networks that span current and future habitats, and identifying routes to maintain connectivity and population genetic diversity in the landscape, as illustrated in the case study of Yosemite toad and Himalayan birds (Chen et al. 2022; Maier et al. 2023). Combined with the emerging concept of conservation macrogenetics (Schmidt et al. 2023), conservationists can aggregate the existing genetic and evolutionary datasets to understand the patterns and processes underlying population genetic diversity and evolution on a large scale, thus supporting genetic diversity conservation policies (CBD 2022). Similarly, systematic management of ecosystems and their phylogenetic diversity could also foster ecosystem resilience against climate change, pests, and diseases outbreak. Again, it is crucial to acknowledge the gaps between current modeling approaches and real-world applications, especially given the long-term scales over which biological consequences unfold. Advances in machine learning have demonstrated its abilities in delineating species and management unit (Pyron 2023), making inference about past evolutionary processes (Schrider and Kern 2016) and generating artificial genomes that inherit complex signals of evolution (Yelmen et al. 2021). Further development in the field could offer robust tools for analyzing large eco-evolutionary datasets, improving predictions and optimizing future-oriented conservation designs. Achieving this requires continuous research and adaptive management that brings together local communities, scientists, and policymakers, with collection of baseline data and field feedback. Such conservation strategies should remain flexible and adaptable to meet future challenges, reflecting the evolving nature of biodiversity.

## Concluding Remarks and Future Prospects

Conservation evolutionary biology, as a unified framework, has the potential to better clarify and guide the goals of biodiversity conservation. By integrating evolutionary principles, it enables a deeper understanding of how evolutionary processes interact with the ecological environment to shape the biodiversity pattern of today and in future, which is fundamental for the preservation and management of biodiversity under rapid environmental change. Various conservation practices have benefited from this holistic view, including defining evolutionarily significant units for conservation, setting conservation priorities for species and ecosystems, managing species interactions and ecosystems systematically, and constructing protected area network for future changes.

To further bridge theory and practice to promote the development of this field, it is crucial to: (i) promote long-term, systematic research on species' spatial, temporal, and evolutionary dynamics under environmental changes by regular monitoring and sampling, in order to capture evolutionary processes across time and space, and to deliver robust and generalizable knowledge for conservation planning; (ii) develop innovative models and decision-making frameworks that incorporate ecological, genomic, and evolutionary data to guide adaptive and predictive conservation practices; (iii) enhance communication, education, and capacity-building in conservation evolutionary biology and actively promote the integration of evolutionary insights into international and national conservation policies and frameworks. Grounding conservation practices in evolutionary basis ensures the maintenance of adaptive potential, evolutionary resilience, and the long-term persistence of biodiversity across all levels over time.

## Data Availability

There is no new data associated with this article.
